# The effect of phospho-peptide on the stability of gold nanoparticles and drug delivery

**DOI:** 10.1186/s12951-019-0522-y

**Published:** 2019-08-19

**Authors:** Zhanwu Hou, Zhen Wang, Run Liu, Hua Li, Zhengyi Zhang, Tian Su, Jeffy Yang, Huadong Liu

**Affiliations:** 1 Center for Mitochondrial Biology and Medicine, The Key Laboratory of Biomedical Information Engineering of Ministry of Education, School of Life Science and Technology, Xi’an Jiaotong University, Xi’an, 710049 China; 2 Schulich Medicine and Dentistry, Western University, London, Canada

**Keywords:** Gold nanoparticles, Phosphotyrosine, Nanoparticle stability, Drug carrier

## Abstract

**Background:**

Gold nanoparticles (AuNPs) have been proposed for many applications in medicine and bioanalysis. For use in all these applications, maintaining the stability of AuNPs in solution by suppressing aggregation is paramount. Herein, the effects of amino acids were investigated in stabilizing AuNPs by rationally designed peptide scaffolds.

**Results:**

Compared to other tested amino acids, phosphotyrosine (pY) significantly stabilized AuNPs. Our results indicated that pY modified AuNPs presented a high level of stability in various solutions, and had good biocompatibility. When a pY-peptide was used in stabilizing AuNPs, the phosphate group could be removed by phosphatases, which subsequently caused the aggregation and the cargo release of AuNPs. In vitro study showed that AuNPs formed aggregation in a phosphatase concentration depending manner. The aggregation of AuNPs was well correlated with the enzymatic activity (R^2^ = 0.994). In many types of cancer, a significant increase in phosphatases has been observed. Herein, we demonstrated that cancer cells treated with pY modified AuNPs in conjunction with doxorubicin killed SGC-7901 cells with high efficiency, indicating that the pY peptide stabilized AuNPs could be used as carriers for targeted drug delivery.

**Conclusion:**

In summary, pY peptides can act to stabilize AuNPs in various solutions. In addition, the aggregation of pY-AuNPs could be tuned by phosphatase. These results provide a basis for pY-AuNPs acting as potential drug carriers and anticancer efficacy.

**Electronic supplementary material:**

The online version of this article (10.1186/s12951-019-0522-y) contains supplementary material, which is available to authorized users.

## Introduction

Gold nanoparticles (AuNPs) have great potential in biological applications such as biosensing, bioimaging, photothermal therapy and cargo delivery [1–4]. Based on the unique optical properties of AuNPs, many colorimetric methods have been established for assaying the different kinds of species like metal ions, proteins, nucleic acids, and cells [5–7]. In addition, the fluorescence resonance energy transfer (FRET) quenching mechanisms of AuNPs makes it useful in the bioimaging system for assaying pH, proteins and DNA [8–10]. Since AuNPs are inert and biocompatible, the aggregation of AuNPs can form highly efficient near-infrared (NIR) photothermal transducers for photothermal therapy [11–13]. In medical research, AuNPs are attractive vectors with high efficiency for delivering genes, drugs or biomolecules to target cells [14–17]. However, AuNPs have a tendency of aggregating easily, particularly in the presence of high salts and biological molecules such as nucleic acids and proteins. Although the aggregation of AuNPs is useful for certain events of biomolecular recognition, AuNPs must be stably dispersed in biological fluids in most applications. Therefore, rational design and modification of AuNPs ligands are very important for further applications.

Up to now, some compounds such as tannic acid, polyvinylpyrrolidone, and thiol-ending polyethylene glycol (SH-PEG) have been used to modify AuNPs to improve their stability, dispersibility, and biocompatibility [18, 19]. Tannic acid is useful for stabilizing high concentrations of AuNPs for long term processes, but it lacks stability in high salt conditions, and tannic acid can be easily replaced by other molecules [20, 21]. Polyvinylpyrrolidone could be strongly bound with AuNPs and improve stability, but it is difficult for further modifications [22]. Although SH-PEG could efficiently improve the stability and dispersibility of AuNPs in aqueous solutions, SH-PEG presents some problems such as low functionality and potential immune reaction after repeated high dosage [23, 24]. Therefore, new hydrophilic molecules are still being explored to overcome the problems of these agents.

Some small molecules and amino acids which contain reactive groups for further modifications, such as cysteine, glutathione and dihydrolipoic acid, are attracting more and more attention for the purpose of stabilizing AuNPs [6, 25, 26]. Herein, the effects of amino acids were investigated in stabilizing AuNPs by rationally designed peptide scaffolds. The peptide scaffolds contain three functional regions: the gold-binding motif at the N-terminus, the non-steric spacer in the middle, and the functional group at the C-terminus. The gold-binding motif consists of a cysteine residue bearing a thiol group to form a covalent linkages between the peptide and AuNP. The middle region has two aminocaproic acid (Ahx) spacers to avoid steric interference. The functional residue was changed to each amino acid with different charge status or chemical structure side chains to investigate their effects on the AuNPs surface. We systematically investigated the stability of AuNPs modified with different peptide ligands and disclosed that only negatively charged ligands stabilized AuNPs in aqueous solution. Peptides with other functional groups caused AuNPs to aggregate. Phosphotyrosine (pY) showed dramatically higher stabilizing ability compared to other amino acids.

Tyrosine phosphorylation is a very common post-translational modification in cells [27, 28]. Abnormal tyrosine phosphorylation is a hallmark of cancer cell [29, 30]. In many types of cancer, significant increases of phosphatases were also observed in tumor tissues, including the SH2 domain-containing protein-tyrosine phosphatase-2 (SHP2) [31, 32]. SHP2 is a positive transducer of growth factor, cytokine, integrin, and hormone signaling pathways which regulates a diverse array of processes; its overexpression is related to many diseases [33–35]. The pY-peptide stabilized AuNPs could be induced to aggregate when their phosphate groups (PO_4_^3−^) are removed by phosphatase. The aggregation of AuNPs may enhance their retention time in cancer cells [36, 37]. Therefore, pY-peptide stabilized AuNPs could be an ideal tunable carrier for cancer therapeutic drugs.

AuNPs based drug delivery systems have received much attention in cancer chemotherapy for their decreased adverse effects and improved therapeutic efficacy [38–40]. pY can be the ideal ligand for stabilizing AuNPs. As a natural product, pY has the inherent properties of being biodegradable, biocompatible, and nontoxic. The reactive carboxyl groups of pY are useful for further chemical modifications. Moreover, the tunable aggregation of pY-AuNPs may enhance the retention of the drug in tumor cells. In this work, pY stabilized AuNPs were used as a nanocarrier for drug delivery and it was highly efficient in killing cancer cells. Herein, we hypothesize that pY peptides could be a novel phosphatase sensitive reagent to stabilize AuNPs. It could be used in the application of targeted drug delivery.

## Results and discussion

### Characterizations of AuNPs

**Fig. 1:**
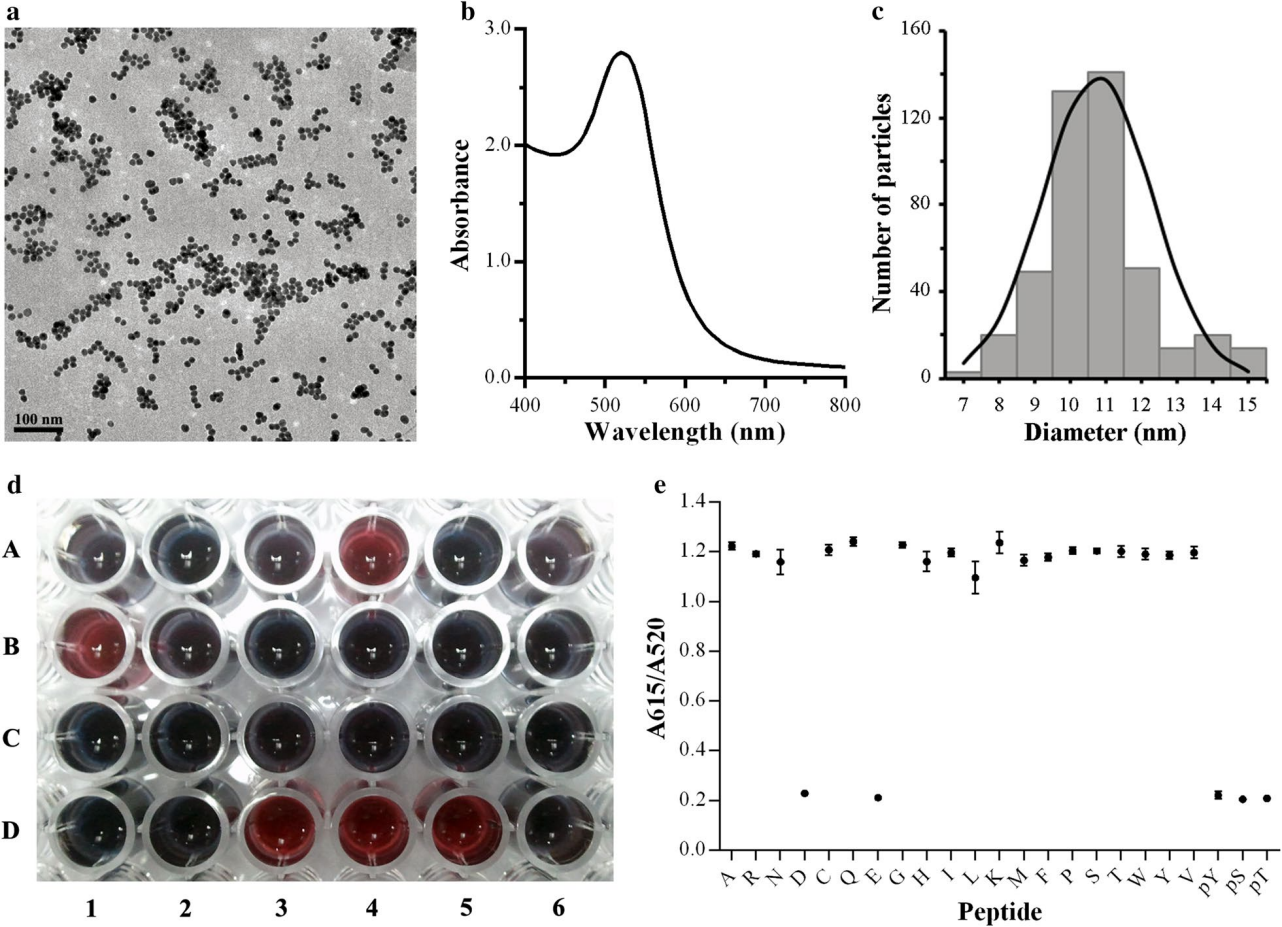
Systematic investigation of different amino acids within scaffold peptides in stabilizing AuNPs. **a** Typical TEM image of AuNPs in water solution (scale bar = 100 nm). **b** UV–Vis absorbance of AuNPs at 400–800 nm with a typical plasmon peak at ~ 520 nm. **c** Histogram depicting the size of 450 particles with mean diameter at 11 nm. **d** Digital image of AuNPs modified by various peptides, blue color indicates aggregated AuNPs and bright red color indicates stabilized AuNPs. **e** The ratio of absorbance at 615 nm to that at 520 nm to show the stability of peptide modified AuNPs

The AuNPs were synthesized using the procedure developed by Turkevich [41]. UV–Vis and transmission electron microscopy (TEM) were used to characterize synthesized AuNPs. TEM analysis revealed AuNPs with a core size of 11 nm (Fig. 1a). The UV–Vis absorbance measurement of AuNPs showed surface plasmon peak bands detectable at 520 nm (Fig. 1b). The size of AuNPs was measured to fit the gaussian distribution with a mean size of 11 nm and diameters were calculated by counting more than 450 of AuNPs (Fig. 1c). The final concentration of synthetic AuNPs was calculated to be 10 nmol/L.

To assess the stability of different amino acids when binding to AuNPs, a series of hybrid nanoparticles were constructed by modifying the gold surface with rationally designed peptide ligands (Additional file 1: Table S1). Once the AuNPs were modified with peptide ligands, the color of AuNPs changed from bright red to dark blue, which indicated serious aggregation of AuNPs. In contrast, the AuNPs reacted with peptide ligands maintained a transparent red color all the time indicating good dispersion of AuNPs (Fig. 1d). The aggregation of AuNPs resulted in spectral changes from 520 nm to longer wavelengths (Additional file 1: Figure S1). Thus the ratio of the absorbance at 615 nm to the absorbance at 520 nm showed the stability of AuNPs (Fig. 1e). A smaller ratio indicated better stability. Above all, it indicated that these peptides with carboxyl and phosphate groups could stabilize AuNPs in physiological ionic solutions, but the other peptides would induce severe aggregation of AuNPs. Carboxyl and phosphate groups provided a negative shell which allowed for the stability of AuNPs [25]. Compared with carboxyl groups, phosphate groups have a higher electronegativity. In addition, a phosphate group contains more potential applications because it could be added onto relevant amino acid residues by kinases and removed by phosphatases in cellular activities. Therefore, the phosphorylated amino acids were used for further investigation.

### Stability of phosphotyrosine-modified AuNPs

**Fig. 2:**
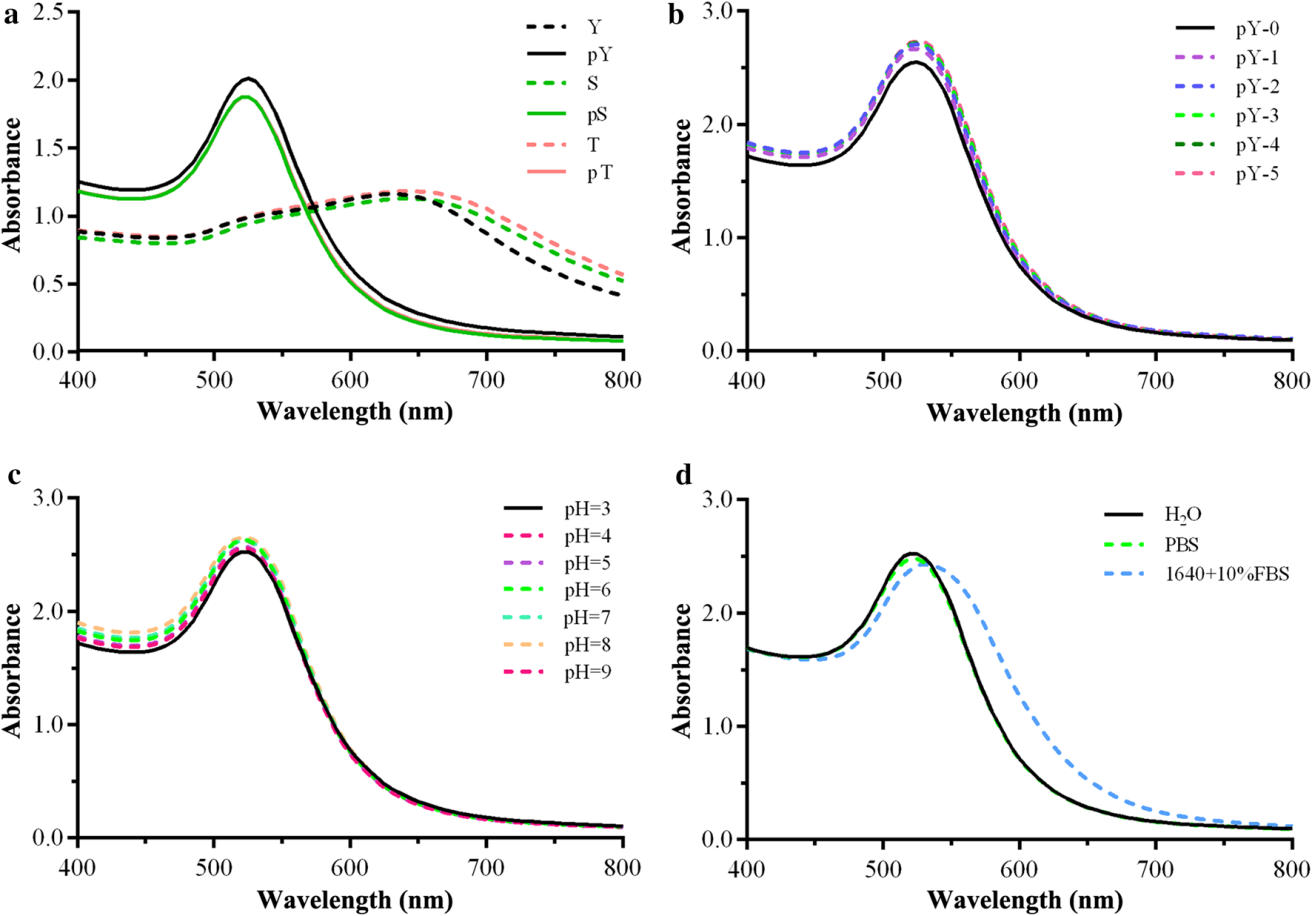
UV–Vis spectra of pY stabilized AuNPs in different conditions. **a** UV–Vis spectra of AuNPs modified by phosphorylated and non-phosphorylated peptides. **b** UV–Vis spectra of AuNPs modified by different length peptides (peptide sequences listed in Additional file 1: Table S2). **c** UV–Vis spectra of C-pY-Ahx modified AuNPs with various pH from 3 to 12. **d** UV–Vis spectra of C-pY-Ahx modified AuNPs incubated in different solutions

Tyrosine, serine and threonine are the common amino acids which can undergo phosphorylation. The peptide scaffolds containing these three amino acids were selected for further testing. As shown in Fig. 2a, peptides without phosphate groups can’t stabilize the AuNPs. This indicated that AuNPs stability was maintained by the presence of the phosphate group. It was supported by the evidence that AuNPs modified with phosphorylated peptides could switch the surface charge via dephosphorylation which induced the aggregation of AuNPs (Figs. 1d and 2a). To check whether the distance between phosphate groups and AuNPs affected its function, several phosphorylated tyrosine peptides were synthesized (Additional file 1: Table S2), and the number of Ahx was used to modulate the distance of negative groups from the Au surface. Under our conditions, the change of the distance had no influence on the colloidal stability (Fig. 2b). However, when the peptide was too long, brush effect may have been another factor to maintain the stability of AuNPs [42], so two amino acid residues were chosen as spacers in further experiments.

The stability of pY coated AuNPs were investigated under various conditions, including solutions of different pH and RPMI-1640 medium with serum. Under pH range from 3 to 12, all the UV–Vis spectra of pY-AuNPs kept a typical plasmon peak at ~ 520 nm, which indicated that no detectable aggregation of AuNPs had happened (Fig. 2c). With the change of the salt concentration, typical plasmon peak (Additional file 1: Figure S2) revealed that pY-AuNPs were well dispersed in high salt concentration solutions. These indicated that pY stabilized AuNPs are tolerant to broad pH values and ion strength ranges. The stability of AuNPs in biological media was also monitored by UV–Vis spectroscopy. When the particles were incubated in RPMI-1640 cell culture medium with 10% FBS, the shape of the UV–Vis spectrum of AuNPs only had a slight change at typical peak (Fig. 2d). All of the evidence implied that the pY-AuNPs had good stability and biocompatibility in biological systems. These characteristics provided confidence for further investigations on pY-stabilized AuNPs.

### The influence of pY containing functional peptides on the stability of AuNPs

**Fig. 3:**
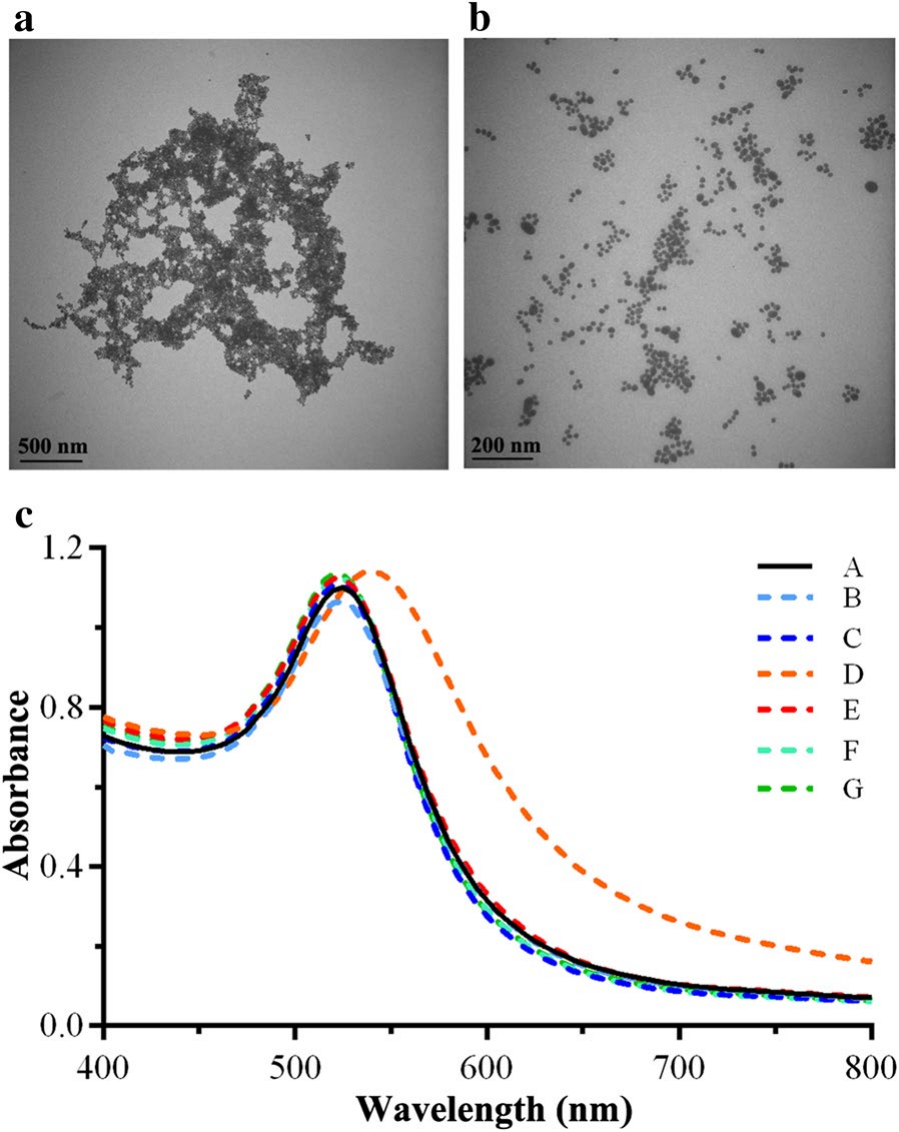
The stability of pY peptide modified AuNPs. **a** Typical TEM image of AuNPs modified with non-phosphorylated peptide in aqueous solutions (scale bars: 500 nm). **b** Typical TEM image of AuNPs modified with phosphorylated peptide in aqueous solutions (scale bars: 200 nm). **c** UV–Vis spectra of AuNPs modified with various phosphorylated peptides (peptide sequences listed in Additional file 1: Table S3)

AuNPs modified with peptides can provide a range of surface functionalities because peptides can provide a valuable resource to control structures and properties of AuNPs [43, 44]. For instance, massive peptide-AuNPs based assays have been developed for detecting different targets including metallic cations, small molecules, nucleic acids, proteins and cells [45]. Cell-penetrating peptides are used to enhance the AuNPs carrier efficient [46]. These technological applications normally require the peptide modified AuNPs to have a high stability and solubility in media. PEG is widely used in enhancing the stability of peptide-AuNPs. However, because of the steric hindrance, PEG could influence the function of peptides [24]. In our work, pY was added into the sequence of the functional peptides to eliminate the steric influence of stabilizing module. The stability of these peptide-AuNPs were monitored by TEM and UV–Vis spectroscopy. As shown in the TEM images, aggregation was observed in AuNPs conjugating with C-Y-Ahx-R-G-D-M-Y-G peptides (Fig. 3a). Interestingly, the AuNPs conjugating with C-pY-Ahx-R-G-D-M-Y-G peptides were well dispersed in PBS (Fig. 3b). It revealed that phosphate groups can reverse the property of the pY-peptide modified AuNPs. The ability of other peptides (Additional file 1: Table S3) in stabilizing the AuNPs were investigated by the UV–Vis. As shown in Fig. 3c, the typical of peak at 520 nm indicated that AuNPs aggregations were inhibited by all of the checked pY-peptides. These implied that the stabilizing ability is in a sequence independent manner, which means pY could be incorporated in various peptides with different functions to stabilize AuNPs.

### Phosphatase regulation of pY-peptide stabilized AuNPs

**Fig. 4:**
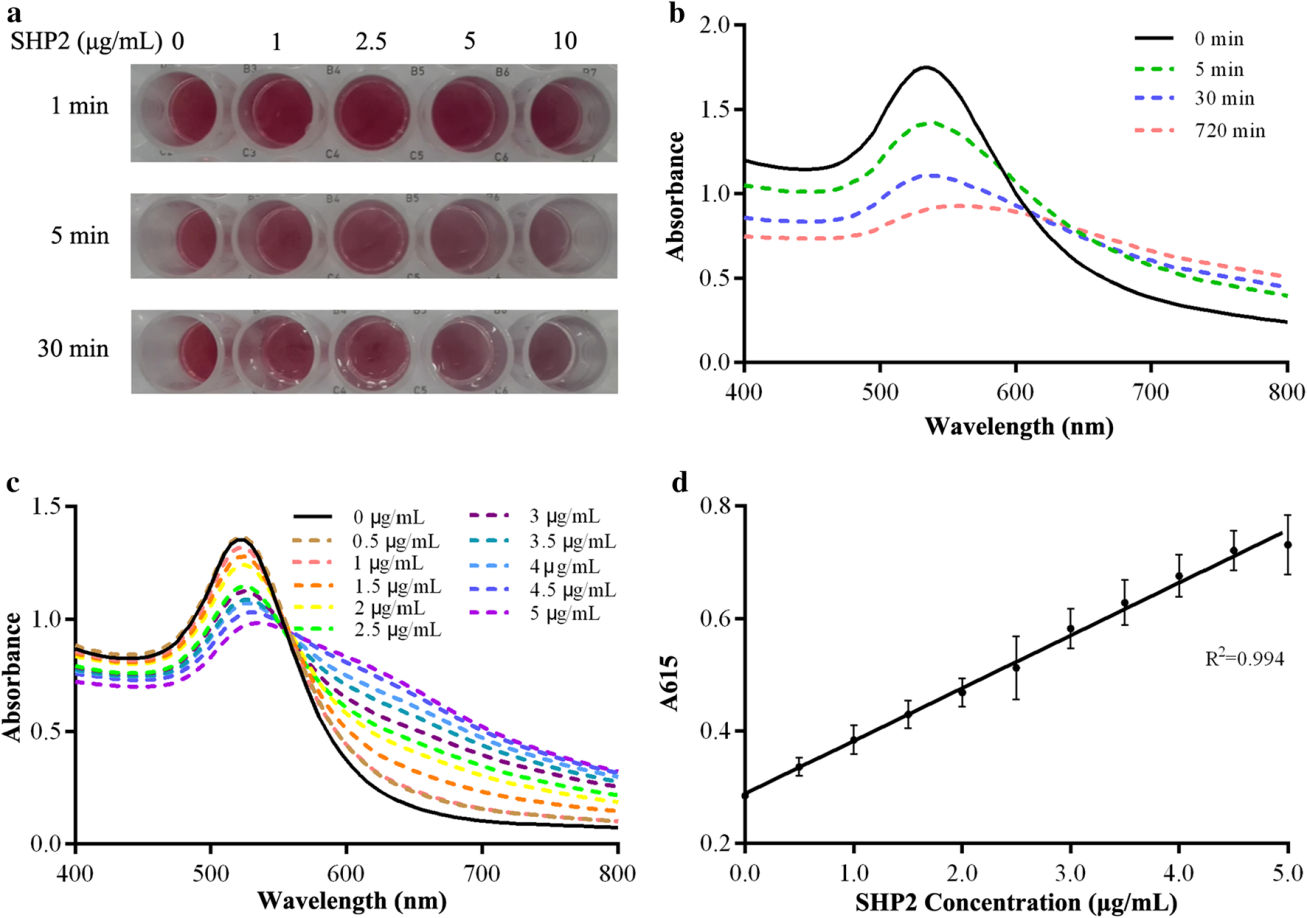
Characterization of SHP2 treated pY-AuNPs. **a** Colorimetric assay of pY-AuNPs treated with SHP2. **b** UV–Vis spectra of pY-AuNPs solutions at different time points after treatment with SHP2. **c** UV–Vis spectra of pY-AuNPs with different SHP2 concentrations. **d** SHP2 concentration versus absorbance at 615 nm of an AuNPs suspension

As shown in Fig. 4a, the solution color of the pY-peptide modified AuNPs remained unchanged in the different time periods, indicating that the pY-AuNPs was stable in PBS. In the presence of the tyrosine phosphatase SHP2, the color of the AuNPs solution changed from pink-red to violet-blue according to the incubation time length. Additionally, the same trend was observed when the SHP2 concentration was increased. For the solution with both high SHP2 concentration and long incubation time, the color was dark due to the severe aggregation. UV–Vis absorbance of the pY-peptide modified AuNPs changed accordingly with an increase in incubation time (Fig. 4b). This also confirmed the SHP2 induced aggregation. There were significant changes in UV–Vis absorbance during incubation after the addition of enzymes. This might be due to the decreased amounts of phosphorylated peptides produced by SHP2. To make sure the aggregation was induced by enzymes, different SHP2 concentrations were tested. Both a decreased absorbance of the plasmon band at 520 nm and an increased absorbance at 615 nm were observed when comparing with enzyme free control (Fig. 4c). As expected, the absorbance shifted to a longer wavelength with an increased SHP2 concentration. Interestingly, a near-linear correlation between the enzyme concentration and the absorbance at 615 nm was observed in the range of 0–5.0 μg/mL at the 5 min time point (Fig. 4d). Herein, we confirmed that pY-peptide-stabilized AuNPs could be dephosphorylated and cause aggregation in an enzyme concentration depending manner.

**Fig. 5:**
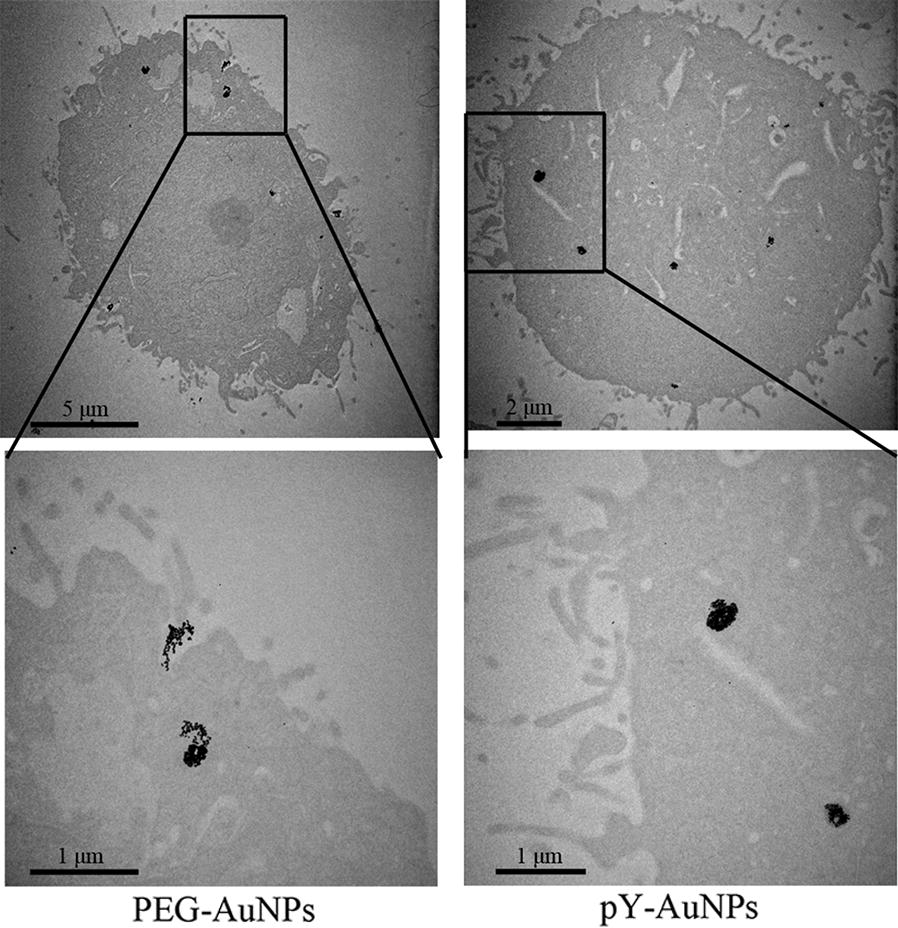
Typical TEM images of SGC-7901 cells incubated with PEG-AuNPs or pY-AuNPs for 12 h

Because the aggregation of AuNPs could increase their tumor retention [36], we wanted to know whether the AuNPs’ aggregations could be formed in living cells by dephosphorylation and thus increasing its tumor retention. To test our hypothesis, SGC-7901 cells were incubated with pY-AuNPs or PEG AuNPs for 12 h. As seen in Fig. 5, TEM images showed that both PEG and pY stabilized AuNPs were able to penetrate the cell membrane and accumulate in cells. However, pY-AuNPs formed tighter aggregations in cells, which was at least partially caused by dephosphorylation. This property provided the possibility of increased the drug retention in tumors through its high SHP2 level.

### Drug targeting delivery of pY-AuNPs

**Fig. 6:**
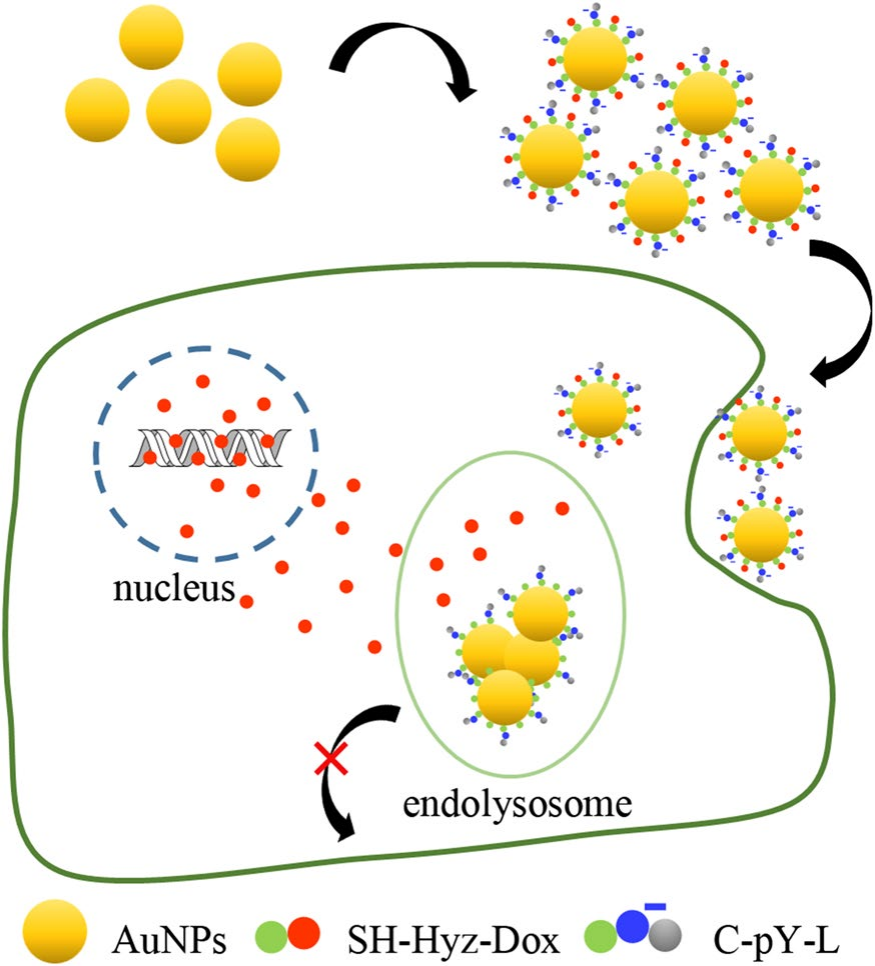
Schematic illustration of pY-AuNPs as a targeted drug delivery system. pY stabilized AuNPs were used as a nano-carrier for drugs (i.e., SH-Hyz-Dox). AuNPs aggregation occurs following dephosphorylation, thus increasing drug tumor retention

**Fig. 7:**
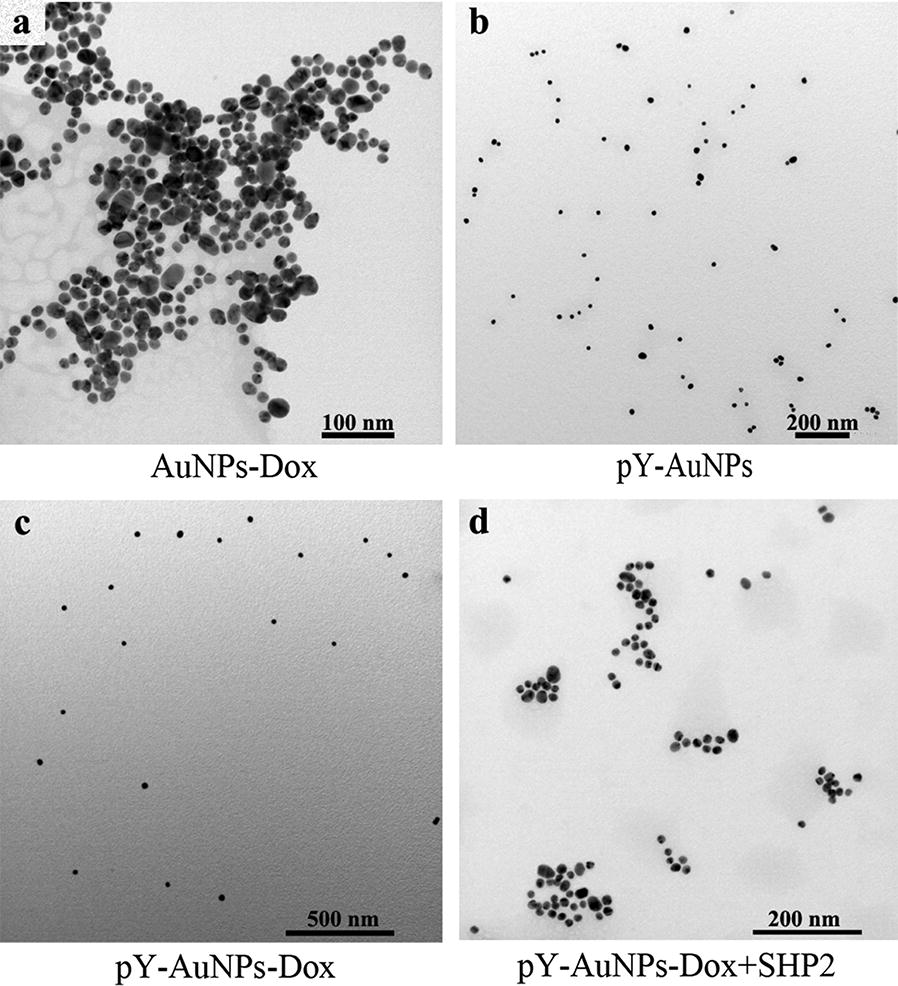
SHP2 triggered aggregation of pY-AuNPs-Dox. TEM images of AuNPs modified with Dox (**a**), pY (**b**) or both (**c**). **d** TEM images of pY-AuNPs-Dox incubated with SHP2 in PBS

Delivery and programmed release of therapeutic materials to specific physiological targets is a key challenge for molecular and macromolecular therapeutics [47]. Several nanocarriers including liposomes, dendrimers, nanocapsules, and metal nanoparticles have been used as promising delivery vehicles [48]. As a nanocarrier, pY is essential in maintaining the dispersion of the AuNPs. In addition, based on the aggregation tunable property of pY-AuNPs, a targeted drug delivery strategy was designed (Fig. 6). To test this strategy, the pY-AuNPs were tested as a carrier for doxorubicin (Dox), one of the most commonly used anticancer drugs but lacking tumor-targeting abilities. To increase its bio-distribution and therapeutic effects, we first synthesized SH-Hyz-Dox which contains a thiol group for binding to the surface of AuNPs (Additional file 1: Figure S3). The pY peptide (C-pY-L) was used to form a complex with AuNPs and SH-Hyz-Dox. As shown in Fig. 7a, AuNPs aggregated after it incubated with SH-Hyz-Dox. However, with the presentence of pY-peptides, the complex showed similar stability (Fig. 7c) as pY-AuNPs (Fig. 7b). These complexes are liable to aggregate again, once very low concentrations of SHP2 were added (Fig. 7d). Additionally, the hydrazone bonds were added between SH– group and Dox, which provided increased drug release.

**Fig. 8:**
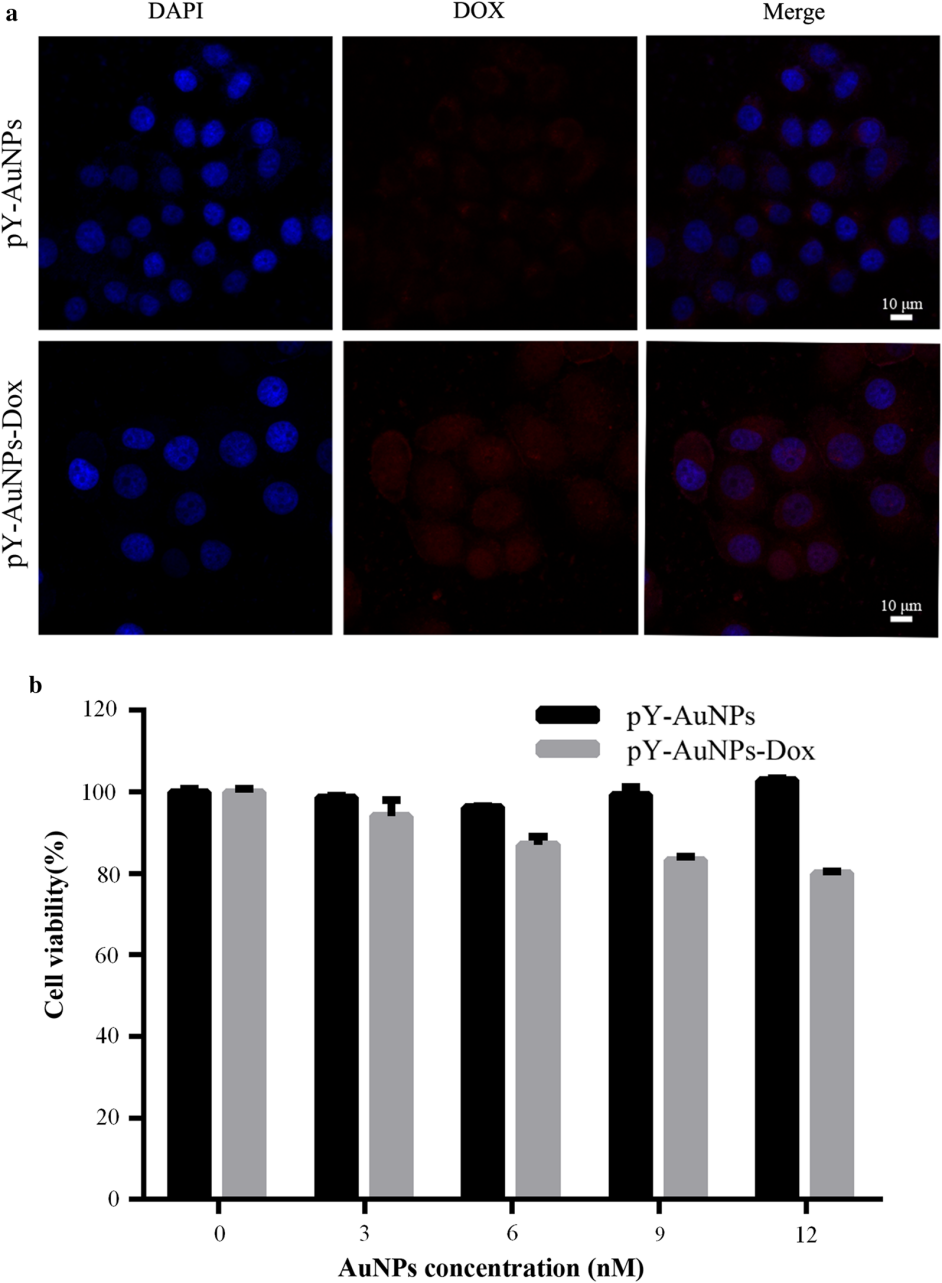
The pY-AuNPs-Dox successfully delivered Dox into cells and caused cytotoxicity. **a** Confocal study of Dox distribution in cells (scale bars: 10 μm). **b** Cell viability assay after pY-AuNPs or pY-AuNPs-Dox treatment for 24 h

SGC-7901 cells are gastric cancer cells which are sensitive to the Dox (Additional file 1: Figure S4). In vitro cytotoxicity of SGC-7901 cells incubated with pY-AuNPs and pY-AuNPs-Dox were evaluated by MTT assay. Confocal laser scanning microscopy (CLSM) images showed that cells incubated with pY-AuNPs-Dox had a stronger red fluorescence than incubation with pY-AuNPs, especially in the nucleus (Fig. 8a). It indicated the uptake of the Dox loaded AuNPs. The overlap of DAPI and Dox signal demonstrated that Dox was released from the AuNPs and entered the nucleus, where it bound with DNA in order to interfere with the transcription process, and thus killed the cancer cells. After a 24 h incubation, the pY-AuNPs and pY-AuNPs-Dox showed the different cytotoxicity based on the AuNPs concentrations (Fig. 8b). As shown in Additional file 1: Figure S5, the deposited number of pY-AuNPs-Dox at different concentrations were calculated by the ISDD platform at Harvard [49]. The results showed the efficiency of pY-AuNPs loading drugs in killing SGC-7901 cells, without showing significant cell toxicity.

## Conclusion

By systematically scanning amino acids, we identified that pY has a strong ability in stabilizing AuNPs. Incorporating pY into Cys-termed peptides, AuNPs were functionalized as a tunable nanocarrier which will be aggregated by phosphatase. Our evidence indicated that pY-AuNPs successfully delivered Dox into SGC-7901 cells without causing significant cell toxicity. To release drugs from lysosome where aggregation accumulated, an acid liable linker was inserted between AuNPs and Dox. The localization of Dox inside the nucleus and the MTT assay showed that pY-AuNPs might be a novel targeting drug delivery system. Since there is a high expression level of SHP2 in tumor tissues, we anticipate that the complex pY-AuNPs-Dox regulated by SHP2 could be optimized to selectively kill cancer cells in further animal studies.

## Experimental section

### Materials and instrumentations

Tetrachloroauric acid (HAuCl_4_·3H_2_O), doxorubicin·HCl (Dox), hydrazine and methyl thioglycolate (MTG) were purchased from Aladdin. Sodium Citrate was purchased from Sinopharm Chemical Reagent Co., Ltd. Fmoc-amine acids, *O*-(Benzotriazol-1-yl)-*N*,*N*,*N*′,*N*′-tetramethyluronium hexafluorophosphate (HBTU), 1-hydroxybenzotriazole (HOBt), and Rink-amine resin were purchased from GL Biochem (Shanghai) Ltd. Ethanol (EtOH), dichloromethane (DCM) and N, *N*-dimethylformamide (DMF) were purchased from CINC High Purity Solvents (Shanghai) Co., Ltd. Piperidine, acetic anhydride and ether were purchased from Xi’an Anbao Chemical Ltd. MTT, triisopropylsilane (TIPS), N, *N*-diisopropylethylamine (DIEA) and trifluoroacetic acid (TFA) were purchased from Sigma. Medium and FBS were purchased from Gibco. SGC-7901 was purchased from TCAA. All water was purified by a Millipore Q system. UV–Vis and MTT were detected by a Molecular Devices Flexstation 3. Transmission Electron Microscopy (TEM) analysis was performed on a Hitachi H-7650 TEM operating in bright field mode.

### Synthesis of water soluble AuNPs

The Turkevich method for the synthesis of a colloidal gold solution uses sodium citrate (Na_3_C_6_H_5_O_7_) reduction of HAuCl_4_ to form AuNPs. Approximately 11 nm AuNPs were synthesized following the steps below. Glassware was cleaned in aqua regia (HCl:HNO_3_ = 1:3) and rinsed with Millipore Q water and then oven-dried before use. Briefly, a solution of HAuCl_4_·3H_2_O (25.0 mM, 4.0 mL) in 95.0 mL deionized water was brought to reflux with stirring. Sodium citrate (40.0 mM, 10.0 mL) was then added quickly. The solution was heated, which resulted in a change in solution color from pale yellow to deep red. After the color had changed, the mixture was stirred for an additional 15 min with the final color being a key characteristic of well-formed 11 nm AuNPs and then allowed to cool down to room temperature. The newly synthesized colloidal gold solution is deemed “as-made” and stored at 4 °C in an opaque glass container. When this protocol is used, the AuNPs consistently have a concentration of 10 nmol/L.

### Peptide synthesis

Peptides were synthesized in our laboratory using Fmoc chemistry. In this method, Rink-amine resin was first swelled in DCM, and then deprotected with 20% piperidine in DMF for 5 min and 20 min, respectively. Next, it was washed thoroughly with DMF-DCM-DMF. 8 aliquots of amino acids and HBTU, HOBt, and 16 aliquots of DIEA were added into DMF for mixing with the resin. The total volume is 2 mL and the reaction would last for 90 min at room temperature. After coupling, the resin was washed thoroughly against DMF-DCM-DMF, then was treated with 20% piperidine in DMF, and washed completely before another amino acid was added to resin. These steps were repeated until all the amino acids were coupled onto the resin. After synthesis, peptides were cleaved from the resin using cocktail buffer (TFA:TIPS:H_2_O = 95:2.5:2.5) for 2 h. Peptides were then precipitated in cold ether and washed with ether for 3 times to remove the Fmoc and other protective groups. Finally, the peptides were dissolved in acetonitrile: H_2_O = 50:50, filtered with 0.45 um filters, freeze-dried and stored at − 20 °C.

### Synthesis the SH-Hyz-Dox

Dox was conjugated onto the surface of AuNPs via the S–Au interaction. The thiol-hydrazine-doxorubicin (SH-Hyz-Dox) synthesis process (Additional file 1: Figure S3a) was based on Santosh Aryal’s work [50]. First, 0.5 µL hydrazine was added into 9 µL MTG at a ratio of 1:10, and the mixture was stirred at 50 °C for 24 h. Then it was mixed with 5.8 mg Dox which was dissolved in 0.5 mL DMSO and reacted in the dark at 50 °C for 24 h. After the reaction was completed, products were confirmed by LC-QTof (Additional file 1: Figure S3b) and the reaction efficiency was monitored by UPLC. The mixture was then purified though a C18 column, freeze-dried and stored at − 20 °C. Prior to use, SH-Hyz-Dox was dissolved in DMSO.

### Peptide conjugation with AuNPs

The peptides bind to AuNPs surface through thiol group. 1 mL AuNPs was incubated with 20 µL peptide. After 3 h incubation at 150 rpm min^−1^, peptide-AuNPs were obtained. Peptide-AuNPs conjugates were further incubated with SH-Hyz-Dox for 3 h at 150 rpm min^−1^, and washed with PBS to obtain the final peptide-AuNPs-Dox product.

### Physicochemical properties of AuNPs and its conjugates

The AuNPs and its conjugates were suspended in Milli-Q water to achieve an appropriate level of scattering. The NPs were characterized using TEM and Flexstation 3 to analyze the structure and UV–Vis. AuNPs were also measured to fit the Gaussian distribution and diameters of the AuNPs were calculated with mean size of 11 nm. The stability of AuNPs under various conditions was tested by UV–Vis spectra collected within a range of 400–800 nm.

### Cellular uptake

The cellular uptake was assessed with CLSM and TEM. For CLSM, SGC-7901cells were seeded in a 12-well plate with cover glass. After the density reached approximately 50–60%, the media was removed and 1 mL/well of pY-AuNPs, and pY-AuNPs-Dox were added. After 24 h incubation, the cells were washed with 1× phosphate buffered saline (PBS) and then fixed with 3.7% formaldehyde in 1× PBS for 10 min at room temperature. Cell were then stain with DAPI for 15 min, and images were acquired using a CLSM 710 (Carl Zeiss, Gottingen, Germany).

### Calculation of the deposited number

The in vitro sedimentation, diffusion and dosimetry (ISDD) platform was used to calculate the deposited number of nanoparticles. The ISDD model inputs were: particle hydrodynamic diameter, 37.8 nm; media height, 3.3 mm; temperature, 310 K; media density, 1.004 g/mL; and viscosity, 0.00074 Pa s.

### Cell culture and cytotoxicity assay

SGC-7901 gastric cancer cells were cultured in RPMI 1640 media supplemented with 10% fetal bovine serum, 100 U/mL penicillin and 100 μg/mL streptomycin. All cells were maintained at 37 °C in a 5% CO_2_ humidified environment. SGC-7901 cells were seeded in 96-well plates at a suitable density. Cells were first cultured for 24 h and then treated with AuNPs conjugates at various concentrations for 24 h. Cell viability was determined using MTT assay. 20 μl MTT solution (0.5 mg/ml) was added to each well following treatment time, and incubated for 4 h at 37 °C. The MTT solution was replaced with 150 μl DMSO. The absorbance at 570 nm for each well was measured on a Molecular Devices Flexstation 3 unit.

## Additional file

**Additional file 1: Table S1.** Functional groups of different amino acids in scaffold peptides. **Table S2.** List of peptides designed by adding different length of spacers between pY and Cys. **Table S3.** List of different functional peptides-AuNPs stabilized by pY. **Figure S1.** UV–Vis absorbance of 24 peptides modified AuNPs. **Figure S2.** UV–Vis absorbance of pY modified AuNPs in different concentrations of buffer. **Figure S3.** Preparation of SH-Hyz-Dox. (a) Synthesis scheme for the preparation of SH-Hyz-Dox. (b) Characterization of SH-Hyz-Dox by LC-QTof. **Figure S4.** Cell viability assay of SGC-7901 cells treated with different concentrations of Dox.** Figure S5.** The deposited number of pY-AuNPs-Dox onto SGC-7901 was estimated using the ISDD model as a function of time.

## Data Availability

All data generated or analysed during this study are included in this published article and its Additional file.

## Abbreviations

AuNPs: gold nanoparticles
pY: phosphotyrosine
FRET: fluorescence resonance energy transfer
NIR: near-infrared
SH-PEG: thiol-ending polyethylene glycol
Ahx: aminocaproic acid
SHP2: SH2 domain-containing protein-tyrosine phosphatase-2
TEM: transmission electron microscope
Dox: doxorubicin
MTG: methyl thioglycolate
DMF: dimethylformamide
DCM: dichloromethane
EtOH: ethanol
TIPS: triisopropylsilane
DIEA: *N*,*N*-diisopropylethylamine
TFA: trifluoroacetic acid
CLSM: confocal laser scanning microscopy
HAuCl4: tetrachloroauric acid
HBTU: *O*-(benzotriazol-1-yl)-*N*,*N*,*N*′,*N*′-tetramethyluronium Hexafluorophosphate
HOBt: 1-hydroxybenzotriazole
