# Supplementary material for: The effect of phospho-peptide on the stability of gold nanoparticles and drug delivery
Source: J Nanobiotechnology. 2019 Aug 19;17:88. doi: 10.1186/s12951-019-0522-y (PMC6699291; doi:10.1186/s12951-019-0522-y)
Supplement: Supplementary file 1 — Additional file 1: Table S1. Functional groups of different amino acids in scaffold peptides. Table S2. List of peptides designed by adding different length of spacers between pY and Cys. Table S3. List of different functional peptides-AuNPs stabilized by pY. Figure S1. UV–Vis absorbance of 24 peptides modified AuNPs. Figure S2. UV–Vis absorbance of pY modified AuNPs in different concentrations of buffer. Figure S3. Preparation of SH-Hyz-Dox. (a) Synthesis scheme for the preparation of SH-Hyz-Dox. (b) Characterization of SH-Hyz-Dox by LC-QTof. Figure S4. Cell viability assay of SGC-7901 cells treated with different concentrations of Dox. Figure S5. The deposited number of pY-AuNPs-Dox onto SGC-7901 was estimated using the ISDD model as a function of time. [file 12951_2019_522_MOESM1_ESM.docx]

Additional file 1

Table S1: Functional groups of different amino acids in scaffold peptides.

Table S2: List of peptides designed by adding different length of spacers between pY and Cys.

Table S3: List of different functional peptides-AuNPs stabilized by pY.

Figure S1: UV-Vis absorbance of 24 peptides modified AuNPs.

Figure S2: UV-Vis absorbance of pY modified AuNPs in different concentrations of buffer.

Figure S3: Preparation of SH-Hyz-Dox. (a) Synthesis scheme for the preparation of SH-Hyz-Dox. (b) Characterization of SH-Hyz-Dox by LC-QTof.

Figure S4: Cell viability assay of SGC-7901 cells treated with different concentrations of Dox.

Figure S5. The deposited number of pY-AuNPs-Dox onto SGC-7901 was estimated using the ISDD model as a function of time.

Table S1: Functional groups of different amino acids in scaffold peptides.

| Name | Number | Sequence(N-C) | End amino acid side chain structure* and property | |
| --- | --- | --- | --- | --- |
| A | A1 | Cys-Ahx-Ahx-Ala |  | Charge: 0  nonpolar |
| R | A2 | Cys-Ahx-Ahx-Arg |  | Charge: +1 |
| N | A3 | Cys-Ahx-Ahx-Asn |  | Charge: 0  polar |
| D | A4 | Cys-Ahx-Ahx-Asp |  | Charge: -1 |
| C | A5 | Cys-Ahx-Ahx-Cys |  | Charge: 0  polar |
| Q | A6 | Cys-Ahx-Ahx-Gln |  | Charge: 0  polar |
| E | B1 | Cys-Ahx-Ahx-Glu |  | Charge: -1 |
| G | B2 | Cys-Ahx-Ahx-Gly |  | Charge: 0  nonpolar |
| H | B3 | Cys-Ahx-Ahx-His |  | Charge: +1 |
| I | B4 | Cys-Ahx-Ahx-Ile |  | Charge: 0  nonpolar |
| L | B5 | Cys-Ahx-Ahx-Tyr |  | Charge: 0  nonpolar |
| K | B6 | Cys-Ahx-Ahx-Lys |  | Charge: +1 |
| M | C1 | Cys-Ahx-Ahx-Met |  | Charge: 0  nonpolar |
| F | C2 | Cys-Ahx-Ahx- Phe |  | Charge: 0  nonpolar |
| P | C3 | Cys-Ahx-Ahx-Pro |  | Charge: 0  nonpolar |
| S | C4 | Cys-Ahx-Ahx-Ser |  | Charge: 0  polar |
| T | C5 | Cys-Ahx-Ahx-Thr |  | Charge: 0  polar |
| W | C6 | Cys-Ahx-Ahx-Trp |  | Charge: 0  nonpolar |
| Y | D1 | Cys-Ahx-Ahx-Tyr |  | Charge: 0  polar |
| V | D2 | Cys-Ahx-Ahx-Val |  | Charge: 0  nonpolar |
| Py | D3 | Cys-Ahx-Ahx-pTyr |  | Charge: -1 |
| Ps | D4 | Cys-Ahx-Ahx-pSer |  | Charge: -1 |
| Pt | D5 | Cys-Ahx-Ahx-pThr |  | Charge: -1 |
| Ahx | D6 | Cys-Ahx-Ahx |  |  |

* The chemical structures of the functional side chains were generated using ChemBioDraw Ultra 12.0

Table S2: List of peptides designed by adding different length of spacers between pY and Cys.

| Peptide | Sequence |
| --- | --- |
| pY-0 | Cys-pTyr-Ahx |
| pY-1 | Cys-Ahx-pTyr |
| pY-2 | Cys-Ahx-Ahx-pTyr |
| pY-3 | Cys-Ahx-Ahx-Ahx-pTyr |
| pY-4 | Cys-Ahx-Ahx-Ahx-Ahx-pTyr |
| pY-5 | Cys-Ahx-Ahx-Ahx-Ahx-Ahx-pTyr |

Table S3: List of different functional peptides-AuNPs stabilized by pY.

| Peptide | Sequence |
| --- | --- |
| A | C-**pY**-Ahx-G-R-V-P-pY-P-R |
| B | C-Ahx-M-I-I-**pY**-R-D-L-I-S |
| C | C-**pY**-Ahx-M-I-I-pY-R-D-L-I-S |
| D | C-Ahx-**pY**-A-R-A-A-A-R-Q-A-R-**pY** |
| E | C-Ahx-R-G-D-M-**pY**-G |
| F | C-**pY**-Ahx-R-G-D-M-Y-G |
| G | C-**pY**-Ahx-R-G-D-M-**pY**-G |





Figure S1: UV-Vis absorbance of 24 peptides modified AuNPs.





Figure S2: UV-Vis absorbance of pY modified AuNPs in different concentration buffer.


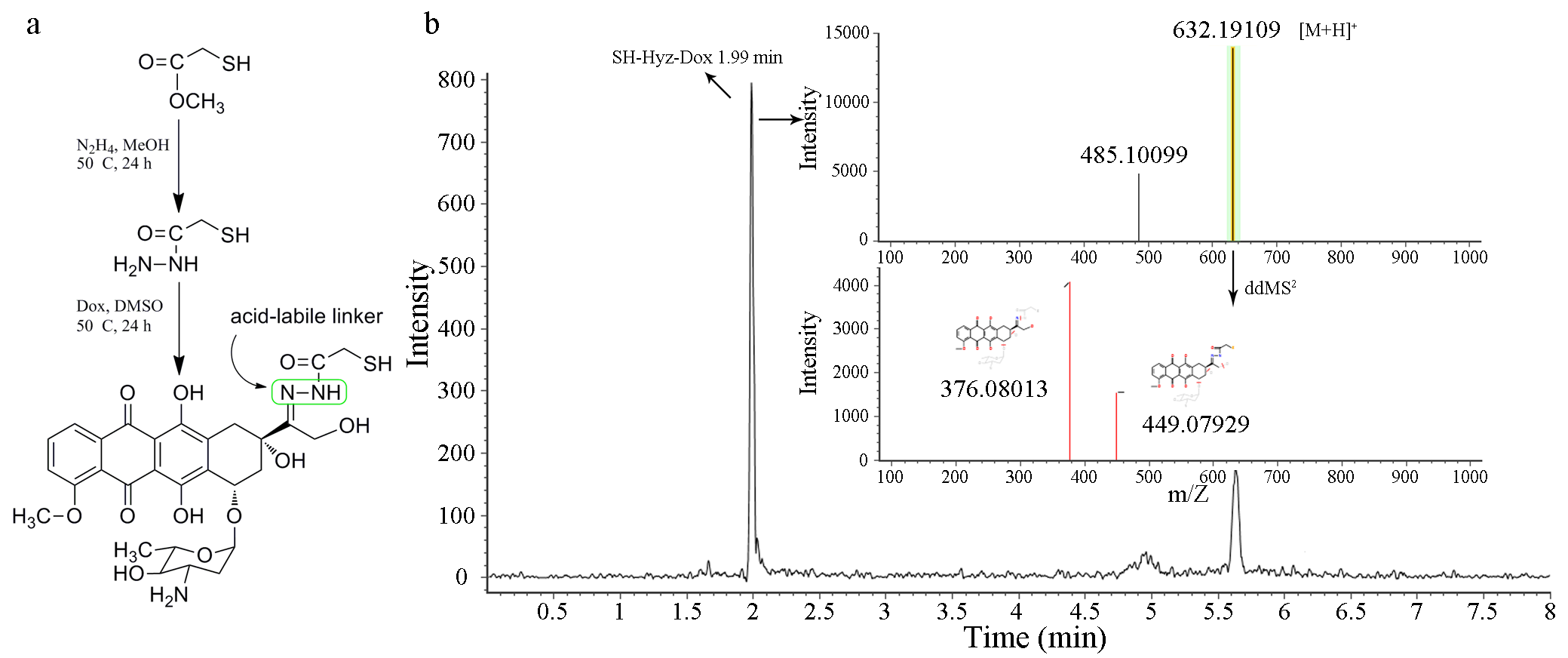


Figure S3: Preparation of SH-Hyz-Dox. (a) Synthesis scheme for the preparation of SH-Hyz-Dox. (b) Characterization of SH-Hyz-Dox by LC-QTof.





Figure S4: Cell viability assay of SGC-7901 cells treated with different concentrations of Dox.





Figure S5. The deposited number of pY-AuNPs-Dox onto SGC-7901 was estimated using the ISDD model as a function of time.
